# An Uncommon Presentation of Hyperthyroidism Can Culminate in Devastating Neurological Consequences: A Case Report

**DOI:** 10.7759/cureus.28424

**Published:** 2022-08-26

**Authors:** Mohammad Abu-Abaa, Shriya Patel

**Affiliations:** 1 Department of Internal Medicine, Capital Health Regional Medical Center, Trenton, USA; 2 Department of Physical Medicine and Rehabilitation, Rowan University School of Osteopathic Medicine, Stratford, USA

**Keywords:** intractable nausea and vomiting, dysphagia, wernicke’s encephalopathy, thyroiditis, hyperthyroidism

## Abstract

Wernicke’s encephalopathy remains largely an underdiagnosed condition. It has two variants: alcoholic and non-alcoholic. A 56-year-old female patient presented with two weeks of persistent nausea, vomiting, and unintentional weight loss. Initial investigations revealed hypercalcemia associated with pancreatitis of biliary origin for which she underwent cholecystectomy as well as thyroiditis resulting in postoperative initiation of methimazole. Persistent symptoms prompted esophagogastroduodenoscopy (EGD), which was unremarkable. She developed diffuse weakness and impaired memory with poor orientation. Magnetic resonance imaging of the brain showed fluid-attenuated inversion recovery (FLAIR) hyperintensity at the central pons and bilateral thalami. Her mental status continued to worsen rapidly within a few days, and she became minimally responsive, hypothermic, and hypotensive; as such, she was intubated for airway protection. Cerebrospinal fluid analysis was unremarkable. She received a thiamine replacement. Repeat MRI after a few days showed improving thalamic hyperintensities with improvement in mentation. This case serves to remind clinicians of the uncommon link between hyperthyroidism and non-alcoholic Wernicke’s encephalopathy (WE).

## Introduction

Wernicke's encephalopathy (WE) is a potentially reversible acute neurological disease, but if missed can result in significant neurological sequelae. Korsafoff syndrome is the late irreversible stage of untreated WE where memory impairment becomes evident with confabulations, providing erroneous memory with no intention to deceive. It results from thiamine deficiency and is classically characterized by the triad of ataxia, confusion, and ophthalmoplegia. The diagnosis remains largely clinical. MRI findings of mainly central hyperintensities are helpful to make the diagnosis but are not required. The vitamin B1 level is limited in specificity but can aid in establishing the diagnosis. WE is most commonly seen in patients with an alcohol use disorder. However, clinicians should remain vigilant about the possibility of non-alcoholic WE. Treatment with thiamine supplementation usually results in the recovery of most of the clinical manifestations. The focus of this report is to demonstrate an uncommon presentation of hyperthyroidism that contributed to the development of WE. This article will be presented on October 24, 2022, at the 6th International Conference on Neurology and Brain Disorders. 

## Case presentation

**Figure 1:**
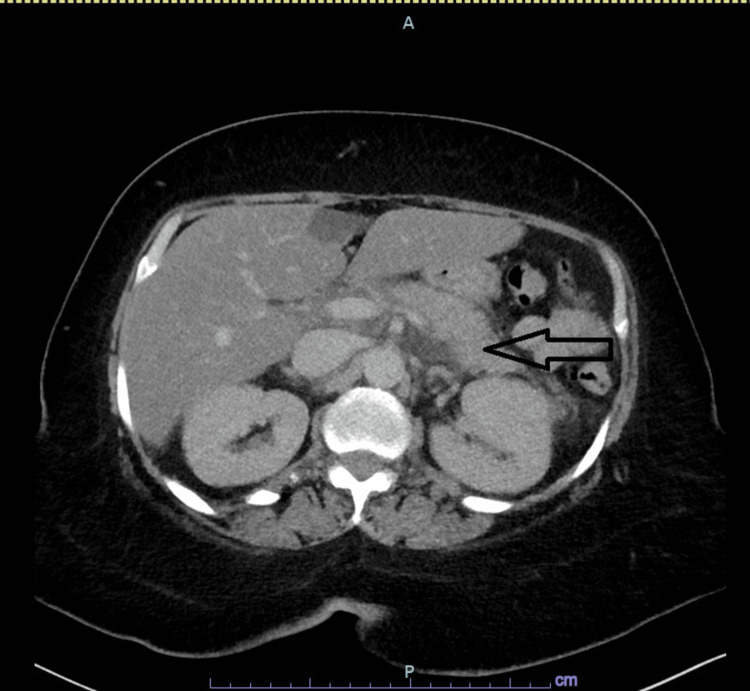
Acute pancreatitis Computed tomography (CT) scan of the abdomen showing pancreatic stranding and peri pancreatic edema compatible with the diagnosis of acute pancreatitis (arrow).

A 56-year-old female with a past medical history of epilepsy presented to the hospital with a two-week history of intractable nausea, emesis almost daily, watery diarrhea, and unintentional weight loss. She also described a history of several months of generalized itching. She denied alcohol intake. Home medications included carbamazepine, phenytoin, and phenobarbital for epilepsy. Computerized tomography (CT) without contrast of the abdomen and pelvis showed moderate pancreatic and peripancreatic edema suggestive of acute pancreatitis (Figure 1).

**Figure 2:**
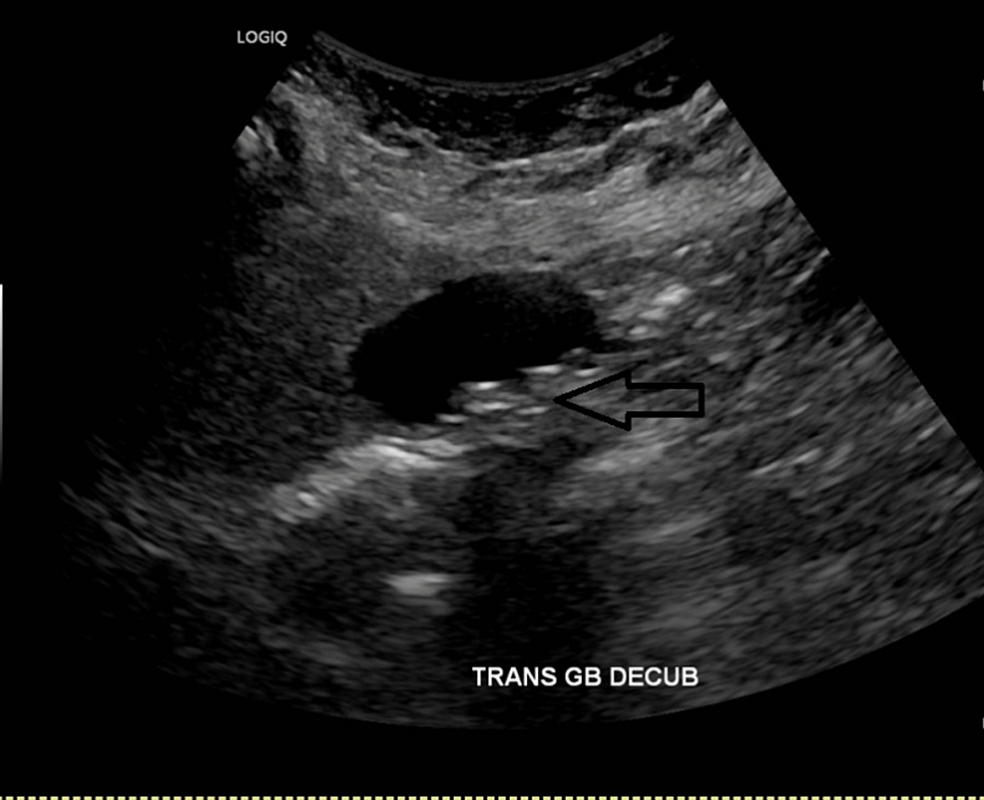
Abdominal ultrasound Abdominal ultrasound showing multiple subcentimetric gallstones without evidence on cholecystitis (arrow).

**Figure 3:**
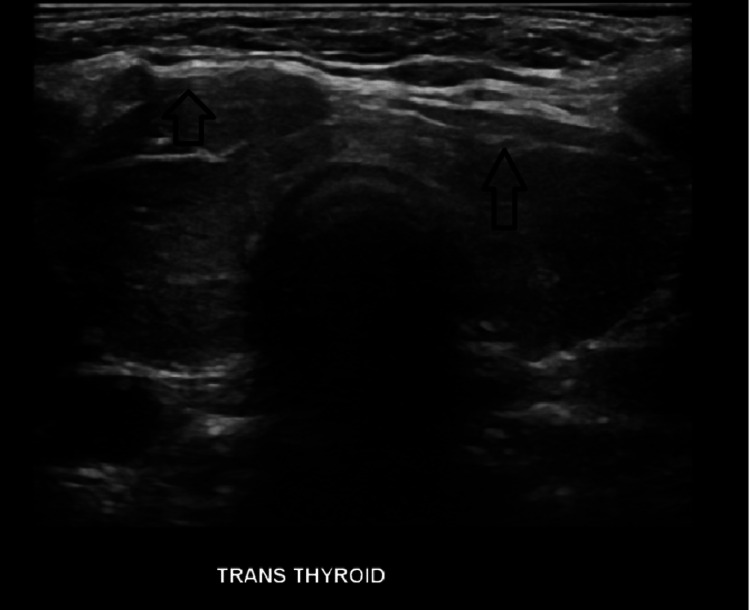
Thyroid ultrasound Thyroid ultrasound showing heterogenous echogenicity of thyroid gland compatible with thyroiditis (arrow).

Abdominal ultrasound (US) showed cholelithiasis but no evidence of cholecystitis (Figure 2). The labs showed hypercalcemia at 12.6 mg/dl and hypokalemia at 2.8 mEq/L. The lipase was also elevated at 677 U/L. As a part of the hypercalcemia workup, T4 free was elevated at 4.8 ng/ml while thyroid-stimulating hormone (TSH) was suppressed below 0.01 mcIU/ml. Thyroid stimulating antibody was high at 39 IU/L (range 0-0.55). Total T3 was normal and the anti-thyroid peroxidase (TPO) antibody was negative. Thyroid US was suggestive of thyroiditis with no thyromegaly or nodules (Figure 3). No features of Grave’s disease were found. Her pancreatitis was presumed to be of biliary origin, and a cholecystectomy was pursued. Aminotransferases were constantly mildly elevated but remained below 100 U/L. Thyroiditis was presumed to be autoimmune in etiology. The creatine kinase (CK) level was normal. Methimazole, 5 mg daily, was started after surgery. Improvement but not complete resolution of the symptoms was achieved and she was discharged home. 

**Figure 4:**
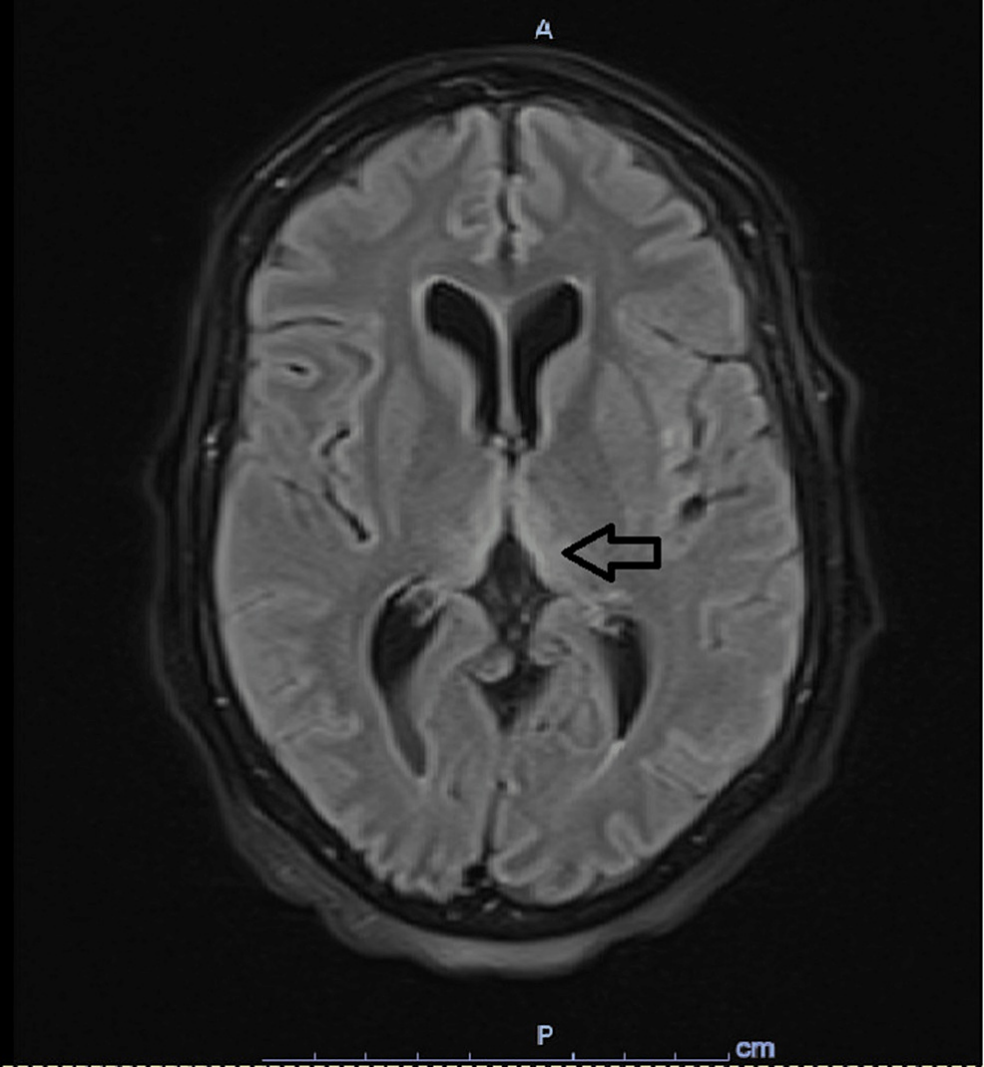
Initial MRI brain MRI of the brain obtained initially showing bilateral thalami hyperintensities (arrow).

Four days later, she was readmitted for the same complaints and inability to comply with her medications secondary to new onset dysphagia to solids and liquids, odynophagia, and choking. Esophagogastroduodenoscopy (EGD) was unremarkable. A physical exam showed an erythematous, beefy tongue suggestive of glossitis. Vitamin B1 and B6 levels were low. However, WE was not considered at the time. She was started on steroids, which she has partially responded to. Video fluoroscopy showed oropharyngeal dysphagia. She missed many doses of methimazole and repeated T4 free was higher at 5.7 ng/dl. She started to have auditory hallucinations and progressive confusion. A neurological exam showed diffuse ⅖ weakness, impaired recent and remote memory, and poor orientation. CT head was unremarkable. The 24-hour electroencephalogram (EEG) was suggestive of encephalopathy with no epileptic spikes. The phosphate level was normal. Methimazole was changed to propylthiouracil, which was administered through a nasogastric tube. Repeat T4 free showed improvement within a few days. Magnetic resonance imaging (MRI) of the brain showed fluid-attenuated inversion recovery (FLAIR) hyperintensity at the central pons and bilateral thalami (Figure 4).

**Figure 5:**
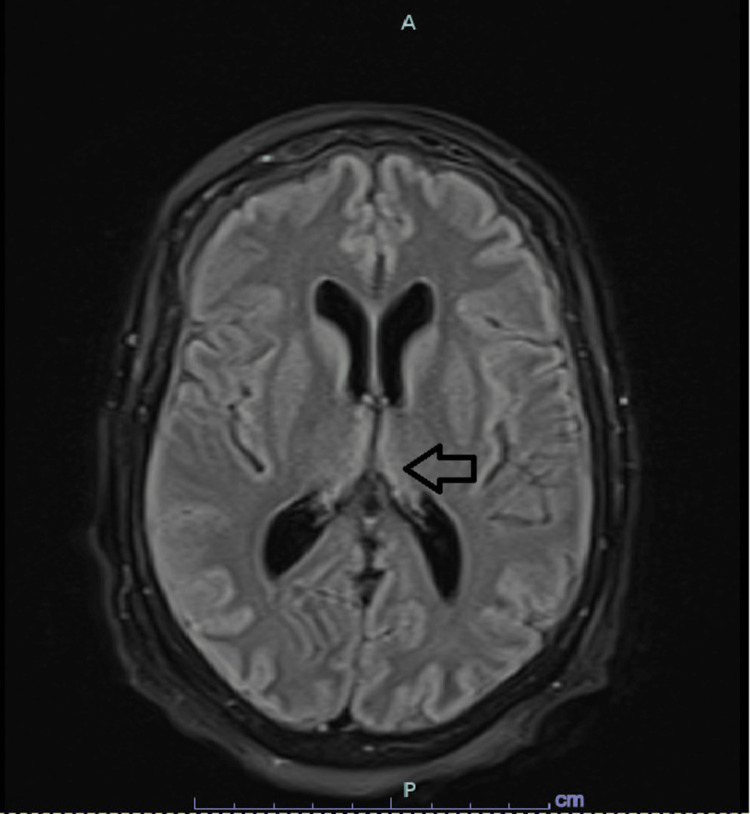
Follow-up MRI MRI of the brain obtained after initial thiamine replacement showing relative improvement in bilateral thalami hyperintensities (arrow).

No disturbances in sodium levels were evident. Her mental status continued to worsen rapidly within a few days and she became minimally responsive, hypothermic, and hypotensive; as such, she was intubated for airway protection. Adrenal insufficiency was ruled out. Cerebrospinal fluid (CSF) analysis was unremarkable. Given the possibility of autoimmune encephalitis, she received empiric plasmapheresis and steroids. Lack of improvement prompted a consideration of WE and thiamine replacement. She received three days of 500 mg intravenous (IV) thiamine, followed by three days of 250 mg IV thiamine, followed by daily 100 mg thiamine replacement. An extensive workup was unyielding for malignancy, syphilis, human immunodeficiency virus (HIV), systemic lupus erythematosus (SLE), Sjogren syndrome, scleroderma, paraneoplastic and autoimmune encephalitis panels. Extubation was feasible after the placement of a tracheostomy and percutaneous endoscopic gastrostomy. Repeat MRI after a few days showed improving thalamic hyperintensities with improvement in mentation (Figure 5).

Over the course of over one month, improvement was evident as she was able to speak with a partial regain of muscle power with a daily thiamine supplement.

## Discussion

The focus of this report is to examine how hyperthyroidism has contributed to the development of WE in this patient. Thiamine can theoretically be depleted within 9-18 days. It is likely that the combination of a hypermetabolic state, which accelerates oxidative phosphorylation and thiamine depletion as thiamine serves as a cofactor for the pyruvate kinase enzyme, and nutritional deficiency accelerated the development of WE in this patient [1,2].

WE, especially non-alcoholic, remains an underdiagnosed condition and it is reported that around 80% of all cases are missed [3]. A common factor among all non-alcoholic causes of WE is nutritional deficiency. It serves the purpose of being vigilant to WE by maintaining a high clinical suspicion of WE whenever risk factors and neurological deficits especially mental status changes co-exist. Risk factors for non-alcoholic WE include hyperemesis gravidarum, chronic kidney disease, thyrotoxicosis, anorexia nervosa, organ transplantation, or total parenteral nutrition [4]. Other risk factors also include magnesium deficiency, malignancies, intensive care unit admission, bariatric surgeries, or a defective soy-based formula, which were described in children and adolescents with WE [5].

The patient's intractable vomiting and poor oral intake were likely multifactorial, including hyperthyroidism, hypercalcemia, and worsened by pancreatitis. Severe intractable nausea and vomiting are uncommon as prominent presentations of hyperthyroidism. Possible mechanisms include gastrointestinal hypermotility, increased beta-adrenergic activity, and impaired neurohormonal regulation [6]. These symptoms are not limited to thyrotoxic storms and can be severe enough to prompt exploratory laparoscopy [7]. These symptoms usually fail to respond to antiemetics but do respond to anti-thyroid medications and steroids. Similarly, dysphagia in this patient is likely multifactorial, including hyperthyroidism and WE. It is rare for hyperthyroidism to cause bulbar muscle weakness and even rarer when it is not associated with other muscle weakness, wasting, and normal CK level [8]. WE can rarely present with dysphagia as motor nuclei of cranial nerves 9th, 10th, and 12th are located in the floor of the fourth ventricle which may be affected by WE [9]. 

The classical clinical triad of WE is in fact uncommon and reported only in 16% of patients [1]. According to autopsy findings, 82% of those with WE had altered mental status ranging from confusion to psychosis. 29% had oculomotor deficits and 23% had ataxia, but 19% had none of the clinical triad [3]. Other uncommon manifestations of WE include seizures, psychosis including hallucinations, hypotension, hypothermia, hearing loss, behavioral changes, and papilledema [10].

The most commonly used clinical criteria are the 2010 European Federation of Neurological Societies (EFNS) criteria, which have a diagnostic sensitivity of 85%. By using these criteria, the diagnosis of alcoholic WE requires two of the following: (a) nutritional deficiency; (b) oculomotor abnormalities; (c) equilibrium disorders; and (d) either an altered mental state or mild memory impairment. These criteria may also be used to diagnose WE in non-alcoholic patients [11]. MRI has a sensitivity of only 53%, and a negative MRI should not preclude the diagnosis [10].

Non-alcoholic WE are more likely than alcoholic WE to have the typical lesions seen on MRI [12]. These typical findings include symmetric hyperintensity of the medial thalami, mammillary bodies, tectal plate, and periaqueductal gray matter, capita of the caudate nucleus, and around the third ventricle. Atypical MRI findings include symmetric hyperintensity of the cerebellum and cerebellar vermis, cranial nuclei of VI, VII, VIII, XII, red nuclei, dentate nuclei, splenium, and cerebral cortex [13].

Patients who did not present in a coma or with cortical damage had resolution of MRI findings and recovery after two weeks to one year of thiamine supplementation [14]. The prognosis of non-alcoholic WE depends on when thiamine supplementation begins. Because non-alcoholic WE is less clinically recognized, it tends to be diagnosed later than alcoholic WE [12], so it is crucial to maintain a high index of suspicion.

## Conclusions

WE, especially the non-alcoholic type, is an under-diagnosed condition. Those with hyperthyroidism have a higher risk of WE secondary to a hyper-metabolic state and accelerated depletion of thiamine. This risk is even higher in those with intractable nausea and vomiting, which are uncommon presentations of hyperthyroidism. Because the diagnosis of WE relies on clinical features and testing with MRI/thiamine level is of limited utility in the acute setting, physicians must maintain a high index of suspicion. Our case adds to a limited body of knowledge suggesting that thyroid IOCs should increase one’s suspicion of WE in patients with altered mental status, especially when associated with poor oral intake.
